# Oral Corticosteroid Bursts and Risk of Serious Adverse Events in Adults With Type 2 Diabetes

**DOI:** 10.1001/jamanetworkopen.2026.35604

**Published:** 2026-09-23

**Authors:** Tsung-Chieh Yao, Chih-Cheng Hsu, Wayne Huey-Herng Sheu, Chi-Shin Wu, Sheng-Mao Chang, Yu-Tung Huang, Yi-Fen Tsai, Hui-Ju Tsai

**Affiliations:** 1 Division of Allergy, Asthma, and Rheumatology, Department of Pediatrics, Chang Gung Memorial Hospital, Taoyuan, Taiwan; 2 School of Medicine, Chang Gung University College of Medicine, Taoyuan, Taiwan; 3 Institute of Population Health Sciences, National Health Research Institutes, Zhunan, Taiwan; 4 Department of Family Medicine, Min-Sheng General Hospital, Taoyuan, Taiwan; 5 Department of Health Services Administration, China Medical University, Taichung, Taiwan; 6 National Center for Geriatrics and Welfare Research, National Health Research Institutes, Huwei Township, Yunlin County, Taiwan; 7 Institute of Molecular and Genomic Medicine, National Health Research Institutes, Zhunan, Taiwan; 8 Division of Endocrinology and Metabolism, Department of Internal Medicine, Taichung Veterans General Hospital, Taichung, Taiwan; 9 Division of Endocrinology and Metabolism, Department of Internal Medicine, Taipei Veterans General Hospital, Taipei, Taiwan; 10 Central Clinic and Hospital, Taipei, Taiwan; 11 College of Medicine, National Defense Medical University, Taipei, Taiwan; 12 Department of Statistics, National Taipei University, Taipei, Taiwan

## Abstract

This cohort study evaluates associations between oral corticosteroid bursts and serious adverse events among patients with type 2 diabetes.

## Introduction

Type 2 diabetes (T2D) affects more than 500 million individuals worldwide and represents a substantial morbidity, mortality, and health care burden.^1,2^ Oral corticosteroids (OCS) are known to disrupt glucose homeostasis. Clinical guidelines recommend close monitoring of blood glucose levels when initiating OCS therapy in patients with T2D.^3^ Although long-term OCS use may cause serious adverse events (SAEs), OCS bursts have been perceived as relatively safe.^4,5^ However, other studies have demonstrated increased risks of rare SAEs in the general population.^6,7,8^

The potential SAEs in patients with T2D, beyond OCS-induced hyperglycemia, remain unknown. We evaluated associations between OCS bursts and SAEs among these patients.

## Methods

This cohort study with a self-controlled case series design used data from the National Health Insurance Research Database (NHIRD), Taiwan, from January 1, 2008, through December 31, 2022 (eMethods in Supplement 1). The study was approved by the institutional review board of the National Health Research Institutes, Taiwan, and followed the STROBE reporting guideline. Data were analyzed from April 1 to November 30, 2025.

The study population included patients with T2D according to *ICD-9-CM* codes 250.x0 or 250.x2 or *ICD-10-CM* code E11 (eTable in Supplement 1). A study flowchart and the study observation periods are provided in eFigures 1 and 2 in Supplement 1. OCS bursts were defined as continuous use of OCS for 14 days or less. The primary outcomes were 5 SAEs, including gastrointestinal tract bleeding, pneumonia, sepsis, heart failure, and fracture, and a negative control outcome, syncope. Conditional Poisson regression models estimated incidence rate ratios (IRRs) with 95% CIs, adjusting for time-varying covariates. Sensitivity analyses were performed. We used the Hochberg procedure to adjust for multiple comparisons, with 2-sided *P* < .05 indicating statistical significance (eMethods in Supplement 1).^9^

## Results

A total of 36 048 participants were eligible for inclusion (mean [SD] age, 61.6 [10.1] years; 52.4% female and 47.6% male). The Table summarizes baseline characteristics of the study population.

**Table.** Baseline Characteristics of Adults With Type 2 Diabetes Who Received OCS Bursts

Characteristic	Study population (N = 36 048)
Age, mean (SD), y	61.6 (10.1)
Sex, No. (%)	
Female	18 875 (52.4)
Male	17 173 (47.6)
OCS burst	
Duration, mean (SD), d	4.5 (2.8)
Duration, median (IQR), d	3 (3-6)
Daily dose, mean (SD), mg	12.7 (7.1)
Daily dose, median (IQR), mg	12 (8-15)
Cumulative dose, mean (SD), mg	56.2 (50.4)
Cumulative dose, median (IQR), mg	45 (30-60)
Prescribing physician specialty, No (%)	
Family practice	8675 (24.1)
Dermatology	8110 (22.5)
Internal medicine	5719 (15.9)
Otolaryngology	4682 (13.0)
Pediatrics	1257 (3.5)
Other	7605 (21.1)
CCI score	
Mean (SD)	2.3 (1.6)
Median (IQR)	2 (1-3)
Category, No. (%)	
0	374 (1.0)
1	14 517 (40.3)
2	8000 (22.2)
3	6278 (17.4)
4	3282 (9.1)
≥5	3597 (10.0)
DCSI score^a^	
Mean (SD)	1.0 (1.3)
Median (IQR)	1 (0-2)
Category, No. (%)	
0	17 211 (47.7)
1	9193 (25.5)
2	5303 (14.7)
3	2292 (6.4)
4	1226 (3.4)
≥5	823 (2.3)
Comorbidity, No. (%)	
Hypertension	23 802 (66.0)
Chronic kidney disease	3886 (10.8)
Cardiovascular disease	2376 (6.6)
Cancer	1574 (4.4)

Abbreviations: CCI, Charlson Comorbidity Index; DCSI, Diabetes Complications Severity Index; OCS, oral corticosteroids.

^a^ Higher scores indicate greater severity of diabetes complications.

Compared with the baseline period, OCS burst initiation was associated with increased risks of 3 SAEs within 6 to 30 days, after adjustment for multiple comparisons (IRR for gastrointestinal tract bleeding, 1.71 [95% CI, 1.52-1.94]; for pneumonia, 2.06 [95% CI, 1.65-2.57]; for heart failure, 1.87 [95% CI, 1.27-2.77]). From 31 to 90 days, increased risks of gastrointestinal tract bleeding (IRR, 1.26 [95% CI, 1.14-1.38]) and fracture (IRR, 1.42 [95% CI, 1.21-1.68]) were observed (Figure). No associations were observed for the negative control outcome. Findings were robust across all sensitivity analyses, including OCS burst use within 30 days, exclusion of death events, alternative definitions of baseline and posttreatment windows, and alternative washout periods (Figure).

**Figure.**
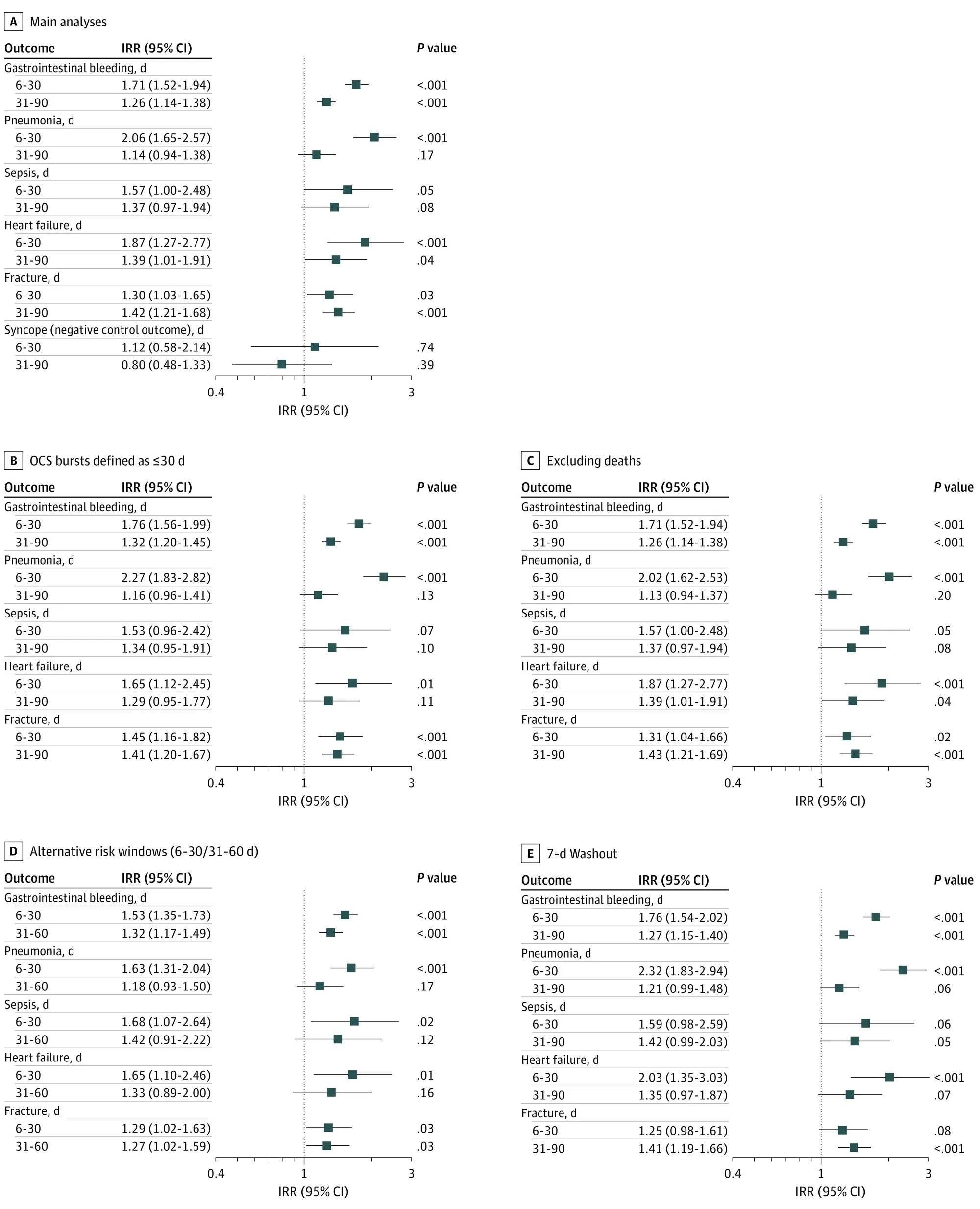
Forest Plots of Main and Sensitivity Analyses of the Association of Oral Corticosteroid (OCS) Bursts With Serious Adverse Events in Patients With Type 2 Diabetes Models were adjusted for time-varying acute conditions (dermatitis and eczema, acute upper respiratory infections, urticarial, acute bronchitis and bronchiolitis, acute sinusitis, acute tonsillitis, pruritus, acute nasopharyngitis, acute laryngitis and tracheitis, and acute pharyngitis, asthma, and allergic rhinitis) and use of nonsteroidal anti*-*inflammatory drugs, proton pump inhibitors, and systemic immunosuppressants. *P* values were calculated after multiple testing correction using the Hochberg procedure. IRR indicates incidence rate ratio.

## Discussion

In this study of 36 048 patients with T2D, OCS bursts were associated with increased risks of gastrointestinal tract bleeding, pneumonia, heart failure, and fracture. The excess risks were most pronounced within the first 30 days following OCS burst initiation, with IRRs ranging from 1.71 to 2.06 compared with the baseline period. These associations were robust in sensitivity analyses. To our knowledge, this study is among the first nationwide investigations to quantify the safety of OCS bursts in patients with T2D, providing evidence of clinically meaningful harms associated with OCS bursts in this high-risk population.

Our findings extend prior work by demonstrating that patients with T2D, who have elevated baseline risks for infection, cardiovascular events, and fracture, experience vulnerability to SAEs of OCS bursts. Studies in Taiwan by Yao et al^7,8^ demonstrated associations between OCS bursts and increased risks of gastrointestinal tract bleeding, sepsis, pneumonia, and heart failure in the general population. We corroborate and extend these findings by focusing on patients with T2D, highlighting that the safety concerns associated with OCS bursts extend to this vulnerable population. From a clinical perspective, these findings underscore the importance of judicious OCS prescribing in patients with T2D, particularly for self-limited conditions for which alternative therapies are available. Although generally perceived as low risk, OCS bursts may contribute meaningfully to morbidity in this high-risk population.

Several limitations should also be noted. First, medication adherence could not be verified. However, any medication nonadherence would likely bias the results toward the null. Second, information on lifestyle factors was unavailable in NHIRD. Although the self-controlled case series design and adjustment for time-varying confounders mitigate confounding, residual unmeasured confounding cannot be completely excluded. Third, outcome misclassification may be a concern, but prior validation studies support the reliability of outcome definitions.^10^ Fourth, caution is warranted when generalizing the findings to other ethnic populations.

In this population-based cohort study of patients with T2D, OCS bursts were associated with increased risks of gastrointestinal tract bleeding, pneumonia, and heart failure within 30 days after initiation. These findings support careful risk-benefit assessment when prescribing OCS bursts in this high-risk population.

## Notes

Supplement 1. **eMethods.** Data, Population, and Analysis **eTable.** *ICD-9-CM* and *ICD-10-CM* Codes Used to Define Type 2 Diabetes, Serious Adverse Events, and the Negative Control Outcome **eFigure 1.** Flowchart of Study Patient Identification **eFigure 2.** Study Design and Definitions of Observation Periods
eReferences

Supplement 2.
Data Sharing Statement
