# Supplementary material for: Oral Corticosteroid Bursts and Risk of Serious Adverse Events in Adults With Type 2 Diabetes
Source: JAMA Netw Open. 2026 Sep 23;9(9):e2635604. doi: 10.1001/jamanetworkopen.2026.35604 (PMC13602001; doi:10.1001/jamanetworkopen.2026.35604)
Supplement: Supplementary file 1 [file jamanetwopen-e2635604-s001.pdf]

Supplementary Online Content

Yao TC, Hsu CC, Sheu WHH, et al. Oral corticosteroid bursts and risk of serious adverse events in adults with type 2 diabetes. *JAMA Netw Open.* 2026;9(9):e2635604. doi:10.1001/jamanetworkopen.2026.35604

eMethods. Data, Population, and Analysis
eTable. *ICD-9-CM* and *ICD-10-CM* Codes Used to Define Type 2 Diabetes, Serious Adverse Events, and the Negative Control Outcome
eFigure 1. Flowchart of Study Patient Identification
eFigure 2. Study Design and Definitions of Observation Periods
eReferences

This supplementary material has been provided by the authors to give readers additional information about their work.

© 2026 Yao TC et al. *JAMA Network Open*.

eMethods. Data, Population, and Analysis

Data Source

This study used data from the entire National Health Insurance Research Database (NHIRD) in Taiwan covering the period from January 1, 2008 through December 31, 2022. The NHIRD is a nationwide medical claims data that contains comprehensive information on the medical and pharmacy claims data for approximately 23.6 million residents, representing 99% of Taiwan's population. Detailed descriptions of NHIRD have been published elsewhere.[1,2] The data were managed, curated, and provided by the Health and Welfare Data Science Center, Ministry of Health and Welfare. The study was approved by the Institutional Review Board of the National Health Research Institutes, Taiwan. The requirement for written informed consent was waived because all data were anonymized and encrypted. This study follows the strengthening the reporting of observational studies in epidemiology (STROBE) reporting guideline for cohort studies.

Study Design and Population

We applied self-controlled case series (SCCS) design to evaluate the association between OCS bursts and the risks of serious adverse events, including gastrointestinal bleeding, pneumonia, sepsis, heart failure and fracture, in patients with T2D. One key advantage of the SCCS design is that the subjects serve as their own controls, a self-controlled approach automatically controlled for unmeasured time-invariant confounding factors in the subsequent analyses.

The study population consisted of adults with T2D, defined as individuals with at least 1 inpatient diagnosis or 2 consecutive outpatient diagnostic records of T2D within one year according to the *International Classification of Diseases, Ninth Revision, Clinical Modification* (*ICD-9-CM*) *codes* 250.x0, 250.x2 or the *International Classification of Diseases, Ninth Revision, Clinical Modification* (*ICD-10-CM*) *code* E11 (**eTable 1**). The

© 2026 Yao TC et al. *JAMA Network Open*.

flowchart of study patient identification and observation periods are provided in **eFigures 1-2**.

Eligible patients were required to meet the following inclusion criteria: 1) age 40 years or older at study entry; 2) no prescriptions for oral, systemic, or topical corticosteroids in the year preceding the baseline period; 3) no recorded diagnoses of the five study outcomes in the year prior to the baseline period; and 4) continuous use of anti-diabetic medications for more than 180 days, including metformin, sulfonylureas, dipeptidyl peptidase 4 inhibitors, thiazolidinediones, sodium-glucose cotransporter 2 inhibitors, α-glucosidase inhibitors, glinides, glucagon-like peptide-1 receptor agonists, or insulin. We further identified patients who had at least one OCS burst prescription and experienced at least one of the investigated serious adverse events during the observational periods and including these individuals in the SCCS analyses.

Exposure Definition and Observation Periods

Prescription records for OCS were ascertained from pharmacy claims within the NHIRD. OCS bursts were defined as continuous use of OCS for 14 days or less. If the gap between two consecutive prescriptions was less than 3 days, the prescriptions were considered as the same OCS course; otherwise, they were treated as separate OCS courses. All corticosteroid agents evaluated in this study were standardized and converted to prednisone-equivalent doses to allow comparison across different formulations.[1]

For each individual, we compared the incidence of serious adverse events during predefined post-treatment risk periods with that during a baseline reference period. The baseline period was defined as 6 to 90 days before initiation of an OCS burst. Two post-treatment periods were specified: 6 to 30 days and 31 to 90 days after OCS burst initiation. A 5-day washout period before and after OCS initiation was applied to minimize potential protopathic bias and misclassification of exposure-related risk windows.

© 2026 Yao TC et al. *JAMA Network Open*.

Study Outcomes

The primary outcomes were five serious adverse events, including gastrointestinal bleeding, pneumonia, sepsis, heart failure and fracture. Outcomes were identified using inpatient claims with the corresponding diagnosis listed as the primary discharge diagnosis, based on *ICD-9-CM codes* prior to 2015 and *ICD-10-CM codes* afterwards (**eTable 1**). Syncope was included as a negative control outcome.

Covariates

Time-varying covariates were adjusted in the analytical models to account for acute changes in health status that could influence both OCS prescribing and the risks of adverse events. These covariates included the 10 most common acute conditions associated with OCS burst prescribing, identified from diagnosis codes recorded at the OCS prescribing visit as well as concomitant medication use, including nonsteroidal anti-inflammatory drugs (NSAIDs), proton-pump inhibitors, and systemic immunosuppressants.

Statistical Analysis

Conditional Poisson regression models were used to quantify incidence rate ratios (IRRs) with 95% confidence intervals (CIs) for each serious adverse event and the negative control outcome. Incidence rates during each post-treatment period were compared with those in the baseline period, with adjustment for time-varying covariates. Analyses were performed separately for each outcome. The list of the adjusted time-varying covariates in the analytical models was described above. We used the Hochberg procedure to adjust for multiple comparisons and control the type I error rate at the predefined 2-sided *P* value of 0.05.[3]

Several sensitivity analyses were performed to assess the robustness and validity of the findings. First, an alternative definition of OCS bursts with a duration of 30 days or less was evaluated. Secondly, analyses excluding individuals who died during any of the observation periods. Third, alternative post-treatment and baseline period definitions were tested: post-

© 2026 Yao TC et al. *JAMA Network Open*.

treatment periods of 6~30 and 31~60 days with a baseline period of 6~60 days prior to OCS initiation. Fourth, instead of a 5-day washout period, a 7-day washout period before and after OCS initiation was applied. We declared levels of statistical significance using a *P* value less than 0.05. All analyses were carried out using SAS version 9.4 (SAS Institute, USA).

© 2026 Yao TC et al. *JAMA Network Open*.

eTable. *ICD-9-CM* and *ICD-10-CM* Codes Used to Define Type 2 Diabetes, Serious Adverse Events, and the Negative Control Outcome

**Diagnosis**	**ICD-9-CM Code**	**ICD-10-CM Code**
Type 2 diabetic mellitus	250.x0, 250.x2	E11
Gastrointestinal bleeding	530.7x, 531.xx, 531.2x, 531.4x, 531.6x, 532.xx, 532.2x, 532.4x, 532.6x, 533.xx, 533.2x, 533.4x, 533.6x, 534.xx, 534.2x, 534.4x, 534.6x, 569.3x, 535.01, 535.11, 535.21, 535.31, 535.41, 535.51, 535.61, 535.71, 537.83, 537.84, 562.02, 562.03, 562.12, 562.13, 569.85, 578.xx	K22.6xxx, K25.xxxx-K28.xxxx, K29.01xx, K29.21xx, K29.31xx, K29.41xx, K29.51xx, K29.61xx, K29.71xx, K29.81xx, K29.91xx, K31.811x, K31.82xx, K52.81xx, K55.21xx, K56.60xx, K57.01xx, K57.11xx, K57.13xx, K57.21xx, K57.31xx, K57.33xx, K57.81xx, K57.91xx, K57.93xx, K62.5xxx, K92.0xxx-K92.2xxx
Pneumonia	481.xx -483.xx, 485.xx - 486.xx	J12.xxxx -J18.xxxx
Sepsis	038.xx, 785.52, 998.02, 995.90- 995.94	A40.xxxx, A41.xxxx, R65.10xx, R65.11xx, R65.20xx, R65.21xx, T81.12XA, T81.12XD, T81.12XS
Heart failure	428.xx	I50.1, I50.9, I50.20-I50.23, I50.30-I50.33, I50.40-I50.43
Fracture	800-829	S02, S12, S22, S32, S42, S52, S62, S72, S82, S92
Syncope	780.2, 992.1	R55

Abbreviations: **ICD-9-CM** = International Classification of Diseases, Ninth Revision, Clinical Modification; **ICD-10-CM** = International Classification of Diseases, Tenth Revision, Clinical Modification.

© 2026 Yao TC et al. *JAMA Network Open*.

eFigure 1. Flowchart of Study Patient Identification

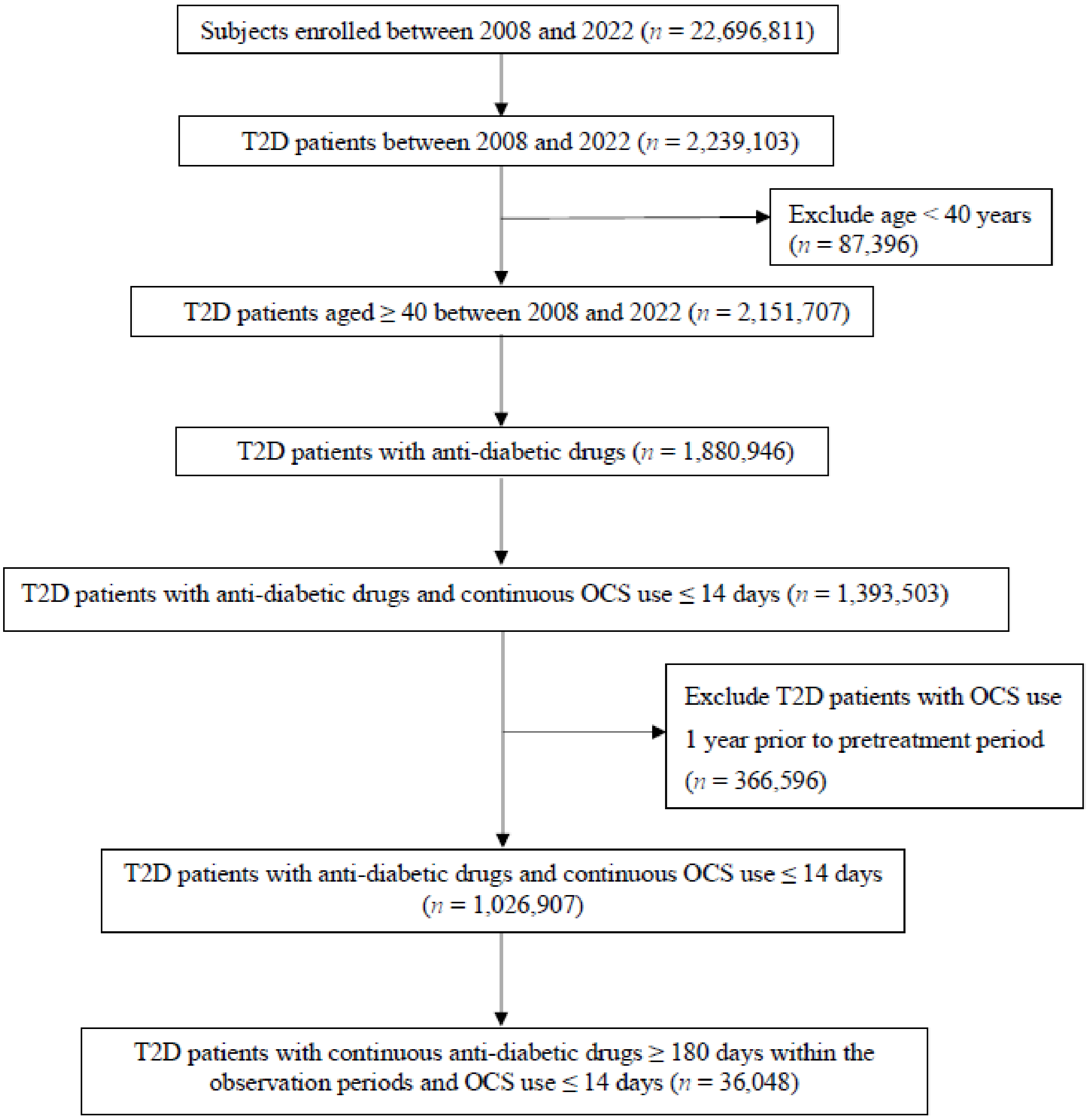

Abbreviations: **T2D**: type 2 diabetes; **OCS**: oral corticosteroids.

© 2026 Yao TC et al. *JAMA Network Open*.

eFigure 2. Study Design and Definitions of Observation Periods

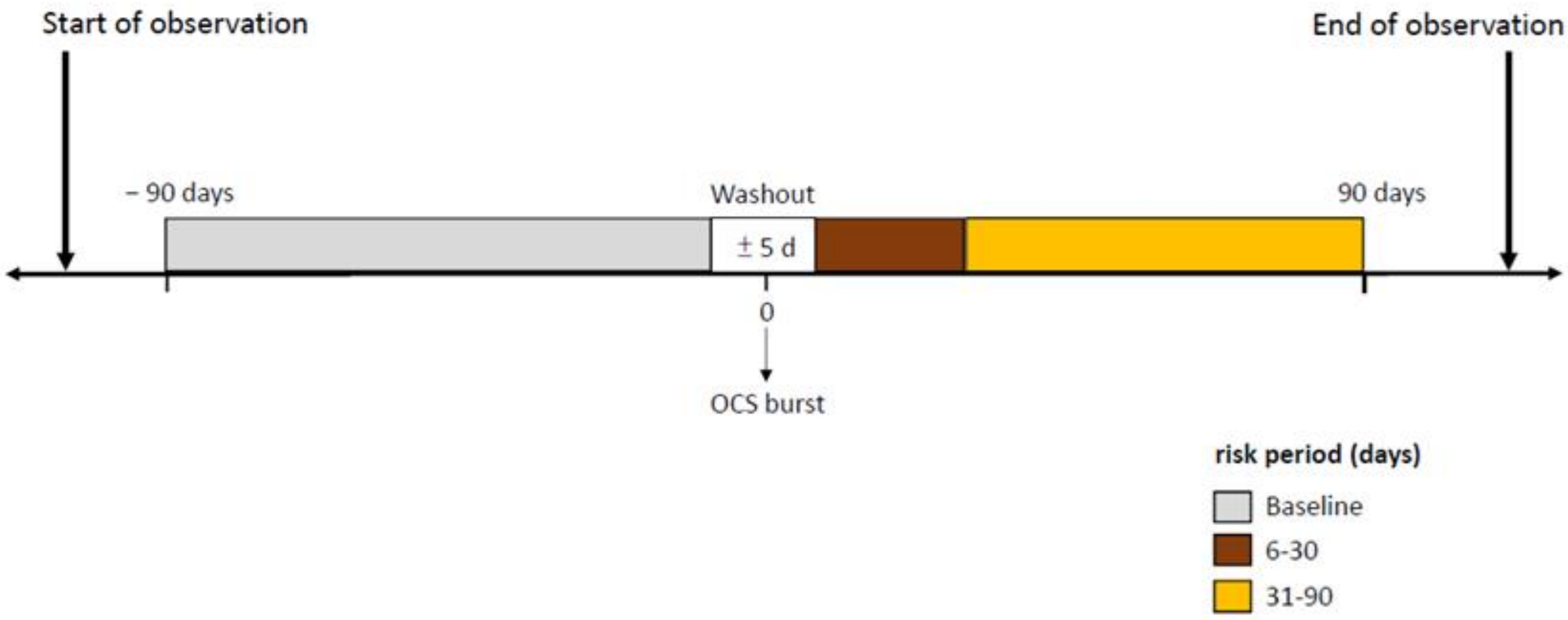

Schematic illustration of the self-controlled case series design and the three observation periods relative to initiation of an oral corticosteroid burst. A 5-day washout period before and after the index date was used to minimize protopathic bias and outcome misclassification.

Abbreviations: **OCS**: oral corticosteroids.

© 2026 Yao TC et al. *JAMA Network Open*.

eReferences

1. Yao TC, Wang JY, Chang SM, Chang YC, Tsai YF, Wu AC, et al. Association of Oral Corticosteroid Bursts With Severe Adverse Events in Children. JAMA Pediatr. 2021;175(7):723-9.
2. Yao TC, Chang SM, Wu CS, Tsai YF, Sheen KH, Hong X, et al. Association between antenatal corticosteroids and risk of serious infection in children: nationwide cohort study. BMJ. 2023;382:e075835.
3. Overbey JR, Wendelberger B, Viele K. The Hochberg Procedure for the Comparison of Multiple End Points. JAMA. 2026;335(15):1351-2.

© 2026 Yao TC et al. *JAMA Network Open*.
