# Supplementary material for: Oral Corticosteroid Bursts and Risk of Serious Adverse Events in Adults With Type 2 Diabetes
Source: JAMA Netw Open. 2026 Sep 23;9(9):e2635604. doi: 10.1001/jamanetworkopen.2026.35604 (PMC13602001; doi:10.1001/jamanetworkopen.2026.35604)
Supplement: Supplementary file 2 [file jamanetwopen-e2635604-s002.pdf]

Data Sharing Statement

Yao. Oral Corticosteroid Bursts and Risk of Serious Adverse Events in Adults With Type 2 Diabetes. *JAMA Netw Open*. Published September 23, 2026.
doi:10.1001/jamanetworkopen.2026.35604

Data
Data available: No
